# Effects of Botanical Blend of Turmeric, Capsicum, and Pepper Extracts on Colostrum and Milk Yield and Quality, Passive Transfer of Immunity, and Performance of Beef Cow–Calf Pairs

**DOI:** 10.3390/vetsci12030250

**Published:** 2025-03-06

**Authors:** Grace H. Jardon, Madison R. Kovarna, Jeff S. Heldt, Emma H. Wall, Ana Clara B. Menezes

**Affiliations:** 1 Department of Animal Science, South Dakota State University, Brookings, SD 57006, USA; grace.jardon@jacks.sdstate.edu (G.H.J.); maidson.kovarna@sdstate.edu (M.R.K.); 2 Selko USA, Indianapolis, IN 46268, USA; jeff.heldt@selko.com; 3 Nutreco Switzerland GmbH, 9320 Arbon, Switzerland; emma.wall@nutreco.com

**Keywords:** average daily gain, immunoglobulins, livestock production, oleoresins

## Abstract

This study evaluated the effects of a botanical supplement (a formulated proprietary blend of turmeric, capsicum, and black pepper oleoresin in a fat carrier; PHYT), fed pre- and post-calving, on colostrum and milk yield and the quality, passive transfer of immunity, and performance of cow–calf pairs. Twenty-three (BW = 532 ± 9.13 kg; age 36 mo) crossbred Angus cows were randomly assigned to three treatment groups: (1) not supplemented (CON, n = 7); (2) supplemented with 250 mg/head/day (PHYT250, n = 8); or (3) supplemented with 500 mg/head/day (PHYT500, n = 8) of the botanical supplement. The cows were individually fed the supplement from 30 days (±6 days) pre-calving to 60 days post-calving. Colostrum was collected on d 0 (pre-suckling), and d 1, d 2, and d 3 post-calving. The total milk from all quarters was collected 45 and 90 d post-calving and at weaning. Colostrum and milk volumes were recorded, and samples were analyzed for percentages of fat, protein, milk urea nitrogen, other solids, and lactose. Colostrum samples were analyzed for concentrations of IgG, IgA, and IgM, and milk samples were analyzed for IgG concentration using radial immunodiffusion. Blood samples were collected from dams and calves, and serum was analyzed for concentrations of IgG (cows and calves) and IgA (calves). Cow and calf BW were measured periodically until weaning. Supplementation with PHYT linearly increased fat in colostrum (CON = 3.29, PHIT250 = 4.23, and PHYT500 = 4.17 ± 0.77%; *p* = 0.05) and IgA in calf serum (96.91, 151.69, and 183.42 ± 29.78 mg/dL for CON, PHYT250, and PHYT500, respectively; *p* = 0.04) and tended to linearly increase concentrations of fat in milk (CON = 3.84, PHYT250 = 4.05, and PHYT500 = 4.71 ± 1.04%; *p* = 0.07), IgG in calf serum (2082.31; 2196.29; and 2577.78 ± 213.08 mg/dL for CON, PHYT250, and PHYT500, respectively; *p* = 0.09), and IgM in colostrum (CON = 179.04; PHYT250 = 170.79; PHYT500 = 218.30 ± 16.08 mg/dL; *p* = 0.09). A quadratic response was observed for calf ADG (*p* = 0.03), where CON (0.99 ± 0.03 kg/d) was less than PHYT250 (1.10 ± 0.03 kg/d), and intermediate values were observed for PHYT500 (1.01 ± 0.03 kg/d). In summary, supplementation with PHYT led to a linear increase in colostrum fat and IgA levels in calf serum. Additionally, supplementation tended to linearly elevate fat concentrations in milk, IgG levels in calf serum, and IgM levels in colostrum. Our results suggest that supplementing beef cows in late gestation and early lactation with 250 or 500 mg/head/d of PHYT improves colostrum quality and calf health and performance. Further investigation is needed to determine both the biological significance and the economic benefits of botanical additives in beef production.

## Simple Summary

Previous studies on turmeric, capsicum, and black pepper have found immune-supporting and anti-inflammatory responses in piglets. However, the effects of this botanical blend on beef calf health remain unknown. Therefore, the objectives of this study were to determine the effects of a botanical supplement pre- and post-calving in beef cows on colostrum and milk quality and the yield, passive transfer of immunity, and cow–calf performance. Three supplementation doses were tested: 0 (CON), 250 (PHYT250), and 500 mg/head/day (PHYT500) from d −30 pre-calving to d 60 post-calving. Supplementation with PHYT resulted in a linear increase in fat in colostrum and IgA in calf serum. Fat concentrations in milk, IgG in calf serum, and IgM in colostrum tended to linearly increase with PHYT. A quadratic response was observed for calf average daily gain (ADG), where CON was lower than PHYT250, and intermediate values were observed for PHYT500. Therefore, supplementing dams with a blend of turmeric, capsicum, and black pepper improved colostrum quality and the passive transfer of immunity to offspring, and also positively impacted calf performance.

## 1. Introduction

The timely intake of high-quality colostrum is crucial for the long-term health, growth, and productivity of calves. Colostrum-derived passive immunity provides essential protection against pathogens and is a key factor in the success of beef cow–calf operations [1]. However, despite the significant role of passive immunity in beef calf health, there remains a notable gap in research specifically focused on improving colostrum quality and passive immunity transfer in this population.

Presently, we are witnessing a growing interest in the use of botanical feed additives in animal feed, including essential oils, spices, herbs, and plant extracts, combined bioactive ingredients and flavoring substances that can potentially improve livestock health and performance [2,3]. In the beef cattle industry, botanicals have been demonstrated to improve dry matter intake and carcass weight [4], enhance ruminal fermentation [5], and reduce inflammation [6]. However, research studies to date have focused on botanical feed additives in the finishing phase. Therefore, the potential benefits of these compounds to the cow–calf sector remain unknown.

Given the crucial role of passive immunity in calf health, investigating the impact of botanical supplementation on colostrum quality and immunity transfer is essential. We evaluated a botanical blend of turmeric, capsicum, and black pepper oleoresins in a fat carrier (PHYT). Earlier studies in lactating sows showed that PHYT supplementation resulted in an increase in sow serum IgG concentration at farrowing and weaning, an increase in sow serum IgA concentration at weaning, and an increase in colostrum IgG concentration [7]. Piglets from supplemented sows also showed greater levels of serum IgA at weaning and increased weaning weights [7]. Additionally, it was found that piglets born from PHYT supplemented sows exhibited a decrease in diarrhea frequency and lower mortality rate compared to piglets from sows not supplemented [7]. Therefore, we hypothesized that the dietary supplementation of beef cows in the pre- and post-partum periods would positively impact colostrum production and improve the concentrations of nutrients and Ig in colostrum. Further, we hypothesized that PHYT could improve the performance of cow–calf pairs. The objectives of this study were to determine the effects of PHYT supplementation pre- and post-calving in beef cows on colostrum and milk quality and yield, passive transfer of immunity, and cow–calf performance.

## 2. Materials and Methods

The animal handling and care practices in this study were approved by the South Dakota State University Institutional Animal Care and Use Committee #2301-015A.

### 2.1. Animals, Experimental Design, and Treatments

Crossbred Angus-based cows (n = 23; 532 ± 9.13 kg; 36 mo of age) originating from the SDSU Cow-Calf Education and Research Facility (CCERF) herd were used in this study. The cows were housed at the CCERF (Brookings, SD) in a dry-lot pen with ad libitum access to feed and water. Thirty days pre-calving (d −30 ± 6), the cows were blocked by weight and randomly assigned to one of three treatments: (1) not supplemented (CON, n = 7); (2) supplemented with 250 mg/head/d (PHYT250, n = 8); or (3) supplemented with 500 mg/head/d (PHYT500, n = 8) of a botanical supplement (a formulated blend of turmeric, capsicum, and black pepper extract [all three ingredients are oleoresins from the fruit] in a fat carrier; Selko^®^ USA, Indianapolis, IN, USA; PHYT). Cows in the PHYT250 and PHYT500 groups were individually fed their supplements in herringbone feeders at 08:00 a.m. daily. The supplements were top-dressed over a premix consisting of corn silage (500–600 g, as-fed) and a mineral and vitamin supplement (113.4 g, as-fed; IntelliBond Mineral, Ralco Nutrition, Marshall, MN, USA). Cows in the CON group were individually fed corn silage along with a mineral and vitamin premix, but without PHYT, at the same supplementation time as previously mentioned. The cows stayed in the feeders for approximately 30 min or until all of the supplement-premix was consumed. The cows received treatments daily from d −30 (±6) to d 60 (±6) relative to calving. All of the cows were group-fed a total mixed ration (TMR) delivered every morning after treatment supplements were fed. Pre-calving, the cows had ad libitum access to round bales of grass hay, and the TMR was made up of corn silage, grass hay, and dried distiller grains with solubles (Table 1). Post-calving, the cows had ad libitum access to round bales of grass hay, and the TMR was made up of corn silage and ground alfalfa hay (Table 1). The post-calving diet was formulated to meet the requirements for early lactation, avoiding nutritional deficiencies (primarily protein and energy), taking into consideration the increase in nutrient demands during this critical window of time (NASEM, 2016). Therefore, high-quality alfalfa hay was included in the diet. The TMR was provided in a bunk feeder in the AM, right after the cows were done consuming their supplements, and the targeted intake was 2.5% of BW. As previously mentioned, both in the pre- and post-calving periods, the cows had ad libitum access to grass hay. The cows and calves remained in the same dry-lot pen up until the end of the supplementation period (d 60 ± 6 relative to calving). Similarly to the cows, all of the calves had ad libitum access to grass hay and TMR. At the end of supplementation, cow–calf pairs were separated by bull and heifer calves and relocated to a pasture with ad libitum access to a mineral and vitamin supplements (Purina^®^ Wind and Rain^®^ All Season Mineral 7.5 Complete, Land O’Lakes, Inc., Arden Hills, MN, USA).

### 2.2. Blood Collections, Calving Procedures, and Colostrum Sampling

For cows, blood samples from the jugular vein were collected in 10 mL serum vacutainer tubes (Becton Dickinson Co., Franklin Lakes, NJ, USA) on d −30, d 0, and d 60 relative to calving. Blood samples of calves were collected on d 0, d 1, and d 60. The samples were centrifuged at 1500× *g* at 4 °C for 20 min and stored in 1.5 mL Eppendorf tubes were stored at −20 °C until further analysis of IgG and IgA concentration via radial immunodiffusion (RID; Triple J Farms, Kent Labs, Bellingham, Washington, DC, USA).

The cows were closely monitored and allowed to calve in their pens. Immediately after calving, cow–calf pairs were moved inside the calving barn for pre-suckling blood and colostrum collections. Colostrum production was determined on a single quarter (left rear) from each cow. Colostrum was collected by hand into a 1000 mL graduated cylinder. The cows were administered 1 mL of oxytocin (20 IU) intramuscularly immediately prior to colostrum collection to induce colostrum letdown. The udder was massaged during colostrum collection until colostrum flow ceased to ensure that the quarter was fully milked out. Colostrum volume was measured, and colostrum was weighed using a bench top scale. Subsamples were collected in 15 mL conical tubes and stored at −20 °C for immunoglobulin (Ig) analysis (IgA, IgG, and IgM). An additional subsample was collected in a 50 mL tube containing a preservative (2-bromo-2-nitropropane-1,3-diol), and stored at 4 °C for further analysis of nutrient composition. Additional colostrum/transition milk samples were collected at 24 h, 48 h, and 72 h after birth (i.e., if a cow calved at 8 am, she was milked at 8 am for the next 3 days). Following pre-suckling sample collections, dams and calves were rejoined in individual maternity pens and observed for nursing within 1 h of sampling procedures. At this time, neonatal calves were assigned a calf vigor score [8] in which calves receive a score of 1 to 5 based on their ability to stand and suckle independently. Calves received vigor scores of (1) normal, vigorous calf; (2) weak calf, but nursed without assistance; (3) weak calf that was assisted to nurse; (4) weak calf that was assisted to nurse but died; or (5) stillborn. The calves were assisted to nurse if needed and pairs were managed in individual maternity pens for at least 24 h after parturition.

### 2.3. Milk Sampling

Milk collections were performed on d 45 and d 90 post-calving, and at weaning (at approximately 7 months of age). Calves were separated from dams the evening before milking (17:00 p.m.), and two hours after initial separation (19:00 p.m.), calves were rejoined for 20 min and allowed to suckle before being separated for the night (approximately 12 total hours of separation). On the following morning (07:00 a.m.), the cows were secured in a chute, administered oxytocin (2 mL; 20 IU) in the jugular vein, and fully milked using a portable milking machine (InterPuls, Albinea, Italy). Prior to the attachment of the milking equipment, all teats were cleaned with a diluted iodine solution and stripped to stimulate milk letdown. The milk was weighed, volume was measured, and samples were stored for nutrient analysis at Dairy Herd Improvement Association Laboratories (Sauk Centre, MN 56378, USA) and for Ig analysis (IgG and IgA) as previously described.

### 2.4. Cow–Calf Bodyweights

The cows were weighed every 14 d, from d −30 to d 60, to monitor body weight gain. The calves were weighed at birth, d 28, d 42, d 56, d 63, and weaning. Body condition scores was assigned throughout this study, on the same time-points mentioned before.

### 2.5. Radial Immunodiffusion Assays

All Ig analyses were conducted using commercial RID test kits (Triple J Farms, Kent Labs, Bellingham, Washington, DC, USA). Cow serum was evaluated for concentrations of IgG and IgA at d −30 (trial initiation), d 0 (calving), and d 60 (trial termination). Calf serum was analyzed for IgG and IgA at d 0 (calving), d 1 (24 h post-calving), and d 60 (trial termination). Further, colostrum samples were analyzed for IgG, IgA, and IgM at d 0 (collected pre-suckling), d 1, d 2, and d 3 post-calving. Milk samples from days 45, 90, and weaning were analyzed for concentrations of IgG.

Approximately 24 h after sample plating, the precipitin rings formed on the RID plates were measured using a digital caliper in which three separate measurements were taken per plated sample and then averaged. All dilutions were performed using a saline solution [0.85% (wt/vol) sodium chloride], prepared by dissolving 8.5 g of sodium chloride in 18 MΩ water, bringing the volume to 1000 mL in a volumetric flask, mixed thoroughly, and subsequently stored at 4 °C. Calf serum, collected 24 h after birth, was subjected to a 2X dilution. All other serum samples originating from both cow and calf were not diluted before IgG and IgA analysis. Colostrum samples were analyzed for IgG using an 8X dilution for d 0 samples. Ultra-low-level IgG plates were used for d 1, d 2, and d 3 colostrum/transition milk samples with a 16X dilution for days 1 and 2 and an 8X dilution for day 3. Colostrum samples were diluted 2X for d 0 and 1 IgA analysis. Colostrum/transition milk IgA concentrations for d 1, 2, and 3 were too low to read at a 1X dilution. Colostrum samples were not diluted for IgM analysis. For milk samples, ultra-low-level IgG RID plates were used, and samples were not diluted. Milk samples were also analyzed for IgA levels, but the concentration of IgA in the milk samples was lower than the minimum concentration needed to form a precipitin ring.

### 2.6. Feed Sampling and Analyses

Samples of feed ingredients were collected weekly throughout the experiment and composited separately for the pre- and post-calving periods. The composited samples was dried in a forced-air ventilation oven at 60 °C for 72 h. Once dried, the samples were ground to a 1 mm particle size using a bench-top cutting mill (Thomas Scientific Wiley Mill, Model 4, Swedesboro, NJ, USA). Feed ingredients were analyzed for DM, crude protein (CP), nitrogen (N), ash, neutral detergent fiber (NDF), and non-fiber carbohydrates (NFC). Dry matter and ash content were determined following AOAC methods: 930.15 and 942.05 [9]. Crude protein and nitrogen were analyzed using the AOAC method 968.06 [9]. Ether extract was determined according to the AOAC method 920.39 [9]. Neutral detergent fiber was analyzed sequentially using thermostable α-amylase without sodium sulfite and without ash correction [10]. Non-fiber carbohydrates (NFCs) were calculated according to the following formula: NFC = 100 − (% CP + % apNDF + % EE + % ash) [11].

### 2.7. Statistical Analyses

The data were analyzed using the MIXED procedure of SAS 9.4 (SAS Inst. Inc., Cary, NC, USA). Body weight; concentration of Ig in serum, colostrum, and milk; and nutrient composition of colostrum and milk were analyzed as repeated measures, with treatment, day, and their interaction as fixed effects, and cow as a random effect. Linear and quadratic contrasts were used to compare CON, PHYT250, and PHYT500. The covariate structures were tested for all variables analyzed, and the structure with the lowest Akaike information criterion/Bayesian information criterion was used. For all analyses, cow was considered the experimental unit, *p*-values ≤ 0.05 were considered significant, and *p*-values of 0.06 to 0.10 were considered tendencies.

## 3. Results

### 3.1. Colostrum and Milk Composition and Volume

There was a linear increase (*p* = 0.05) in fat content in colostrum in response to PHYT (Table 2). A tendency (*p* = 0.09) for a quadratic effect was observed for protein, where concentrations were greater for PHYT500 (8.24 ± 0.32%) compared to CON (7.80 ± 0.34%) and PHYT250 (7.33 ± 0.32%). A main effect of day (*p* ≤ 0.02) was observed, with protein being greater on day 0 compared to all other days, and other solids and lactose being greater on d 3 compared to all other days. The volume of colostrum was not affected by treatment, day, or their interaction.

Milk composition and volume were not affected (*p* ≥ 0.14; Table 3) by treatment × day interactions. Fat concentration tended to linearly increase (*p* = 0.07) in response to PHYT (3.84 ± 0.35, 4.05 ± 0.30, and 4.71% ± 0.30 for CON, PHYT250, and PHYT500, respectively), while MUN tended to linearly decrease (*p* = 0.06) in response to PHYT. Concentrations of protein were greater (3.79%; *p* < 0.01) at weaning, followed by 45- and 90- days post-calving (3.24 and 3.15%, respectively). Concentrations of lactose and other solids were greater (*p* < 0.01) on d 45 followed by d 90 and weaning. The volume of milk was greater (*p* < 0.01) on d 45 and 90 compared to weaning.

### 3.2. Immunoglobulin Concentrations in Serum, Colostrum, and Milk

**Figure 1:**
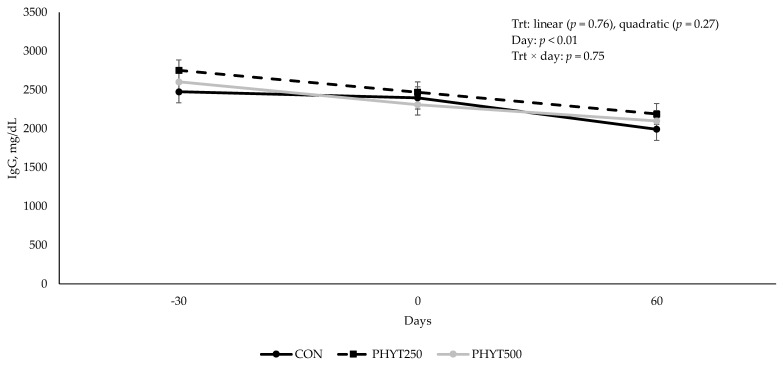
Effects of a botanical blend on immunoglobulin G (IgG, mg/dL) concentration in cow serum. CON, not supplemented; PHYT250, supplemented with 250 mg/head/d (n = 8); PHYT500, supplemented with 500 mg/head/d (n = 8). Supplements were a formulated blend of turmeric, capsicum, and black pepper extract in a fat carrier. Treatments were applied 30 days pre-calving up to 60 days post-calving. Samples were collected 30 days pre-calving (d −30), at calving (d 0), or 60 d post-calving (d 60).

**Figure 2:**
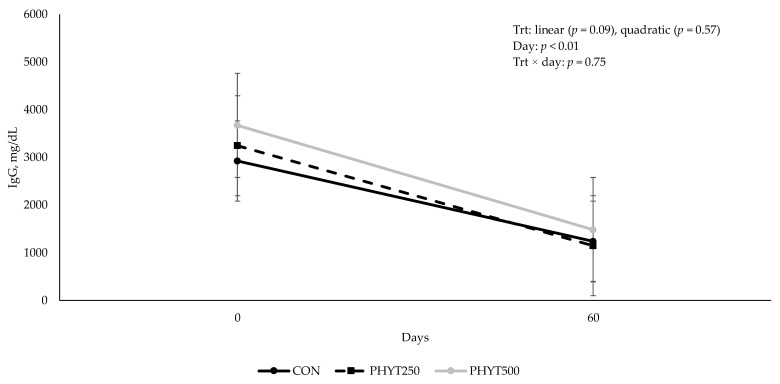
Effects of a botanical blend on immunoglobulin G (IgG, mg/dL) concentration in calf serum. Treatments were applied to dams—cows received treatments from 30 d pre-calving up to 60 d post-calving. CON, not supplemented (n = 7); PHYT250, supplemented with 250 mg/head/d (n = 8); PHYT500, supplemented with 500 mg/head/d (n = 8). Supplements were a formulated blend of turmeric, capsicum, and black pepper extract in a fat carrier. Blood samples on d 0 were collected 24 h after birth to guarantee calves had ingested colostrum.

**Figure 3:**
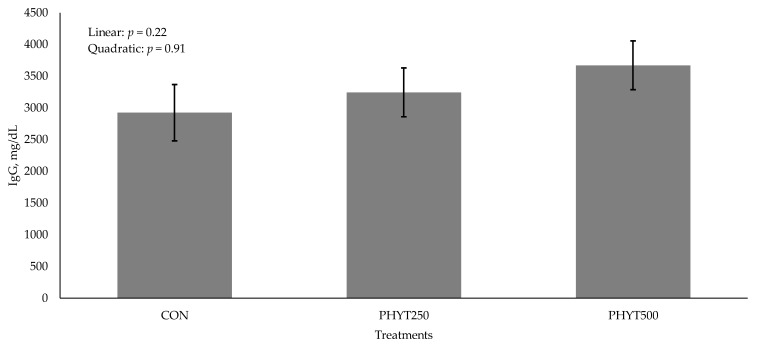
Effects of botanical blend on immunoglobulin G (IgG, mg/dL) concentration in calf serum 24 h after birth. Treatments were applied to dams—cows received treatments from 30 d pre-calving up to 60 d post-calving. CON, not supplemented (n = 7); PHYT250, supplemented with 250 mg/head/d (n = 8); PHYT500, supplemented with 500 mg/head/d (n = 8). Supplements were a formulated blend of turmeric, capsicum, and black pepper extract in a fat carrier.

**Figure 4:**
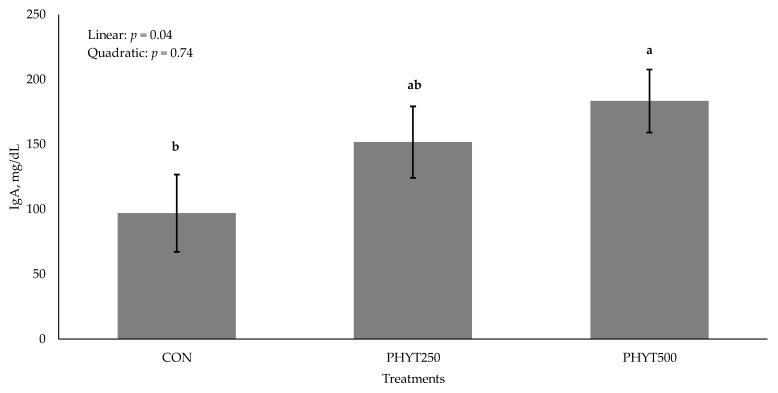
Effects of a botanical blend on immunoglobulin A (IgA, mg/dL) concentration in calf serum 24 h after birth. Treatments were applied to dams—Cows received treatments from 30 d pre-calving up to 60 d post-calving. CON, not supplemented (n = 7); PHYT250, supplemented with 250 mg/head/d (n = 8); PHYT500, supplemented with 500 mg/head/d (n = 8). Supplements were a formulated blend of turmeric, capsicum, and black pepper extract in a fat carrier). ^a–b^ Means with different superscripts differ significantly (*p* ≤ 0.05).

Concentrations of IgG in cow serum (Figure 1) decreased (*p* < 0.01) from d −30 pre-calving to d 60 post-calving. As supplementation dose increased, there was a tendency for a linear increase (*p* = 0.09) in IgG concentration in calf serum (2082 ± 213.08, 2196.29 ± 184.53, and 2577.78 ± 0.30 mg/dL for CON, PHYT250, and PHYT500, respectively; Figure 2). Further, calf serum IgG concentrations decreased from d 0 (24 h after birth) to d 60 post-calving (*p* < 0.01). When analyzing the single time-point of 24 h after birth, we did not observe significant differences between treatments (linear: *p* = 0.22; quadratic: *p* = 0.91; Figure 3). Concentrations of IgA in calf serum, collected 24 h after birth, linearly increased (*p* = 0.04) with PHYT, with the greatest values observed for PHYT500 (96.91 ± 29.78; 151.69 ± 27.57, and 183.42 ± 24.32 mg/dL for CON, PHYT250, and PHYT500, respectively, Figure 4).

**Figure 5:**
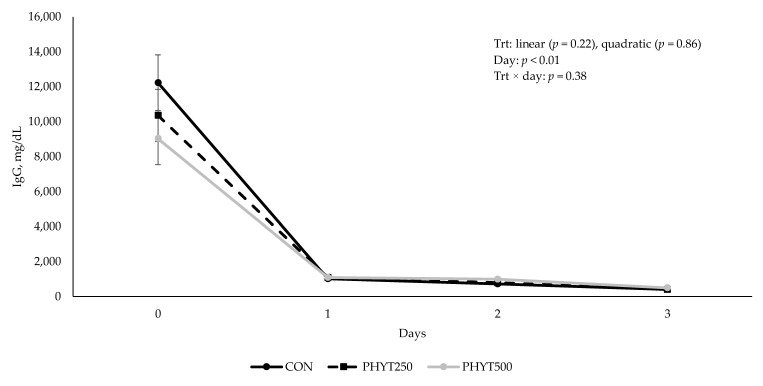
Effects of a botanical blend on immunoglobulin G (IgG, mg/dL) concentration in colostrum and transition milk. CON, not supplemented (n = 7); PHYT250, supplemented with 250 mg/head/d (n = 8); PHYT500, supplemented with 500 mg/head/d (n = 8). Supplements were a formulated blend of turmeric, capsicum, and black pepper extract in a fat carrier. Treatments were applied 30 days pre-calving up to 60 days post-calving. Samples were collected on d 0 (birth), 1, 2, and 3.

**Figure 6:**
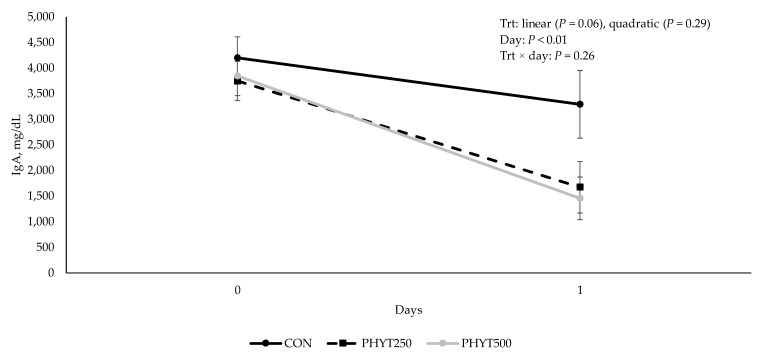
Effects of a botanical blend on immunoglobulin A (IgA, mg/dL) concentration in colostrum. CON, not supplemented (n = 7); PHYT250, supplemented with 250 mg/head/d (n = 8); PHYT500, supplemented with 500 mg/head/d (n = 8). Supplements were a formulated blend of turmeric, capsicum, and black pepper extract in a fat carrier. Treatments were applied 30 days pre-calving up to 60 days post-calving. Samples were collected on d 0 (birth) and 1.

**Figure 7:**
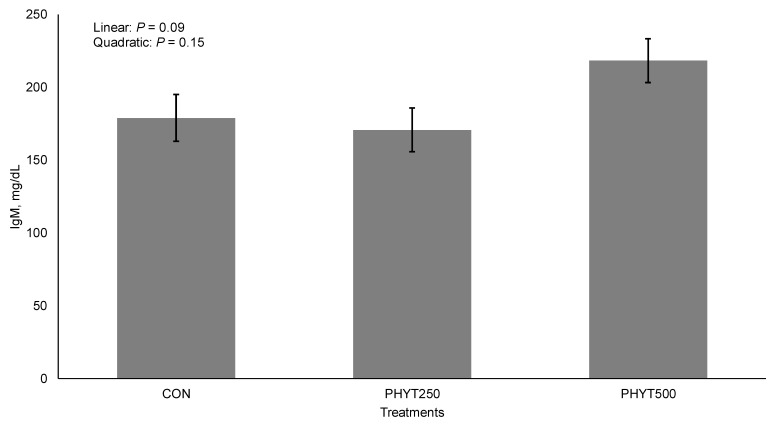
Effects of a botanical blend on immunoglobulin M (IgM, mg/dL) concentration in colostrum. CON, not supplemented (n = 7); PHYT250, supplemented with 250 mg/head/d (n = 8); PHYT500, supplemented with 500 mg/head/d (n = 8). Supplements were a formulated blend of turmeric, capsicum, and black pepper extract in a fat carrier. Treatments were applied 30 days pre-calving up to 60 days post-calving. Samples were collected on d 0 (birth).

For colostrum, no treatment × day interaction (*p* ≥ 0.26) or main effect of treatment (*p* ≥ 0.12) was observed for IgG (Figure 5). Colostrum IgA concentrations tended to be higher in the control group, showing a linear effect (*p* = 0.06; Figure 6). However, as expected, IgG and IgA concentrations were greater (*p* < 0.01) on d 0 compared to all other days. Further, we observed a tendency for a linear increase (*p* = 0.09; Figure 7) in concentrations of IgM in response to PHYT.

**Figure 8:**
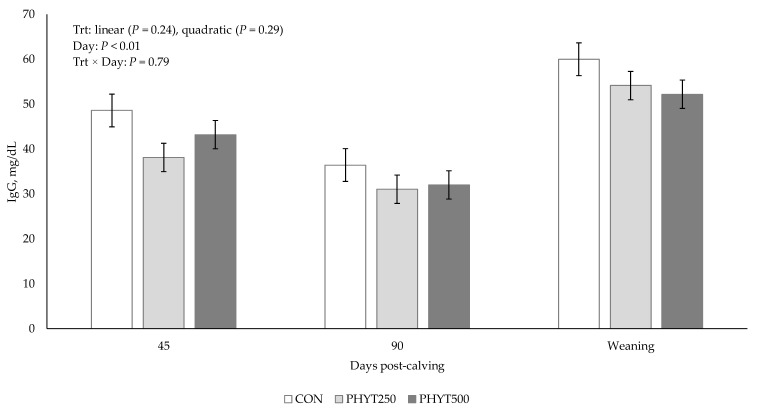
Effects of a botanical blend on immunoglobulin G (IgG mg/dL) concentration in milk. CON, not supplemented (n = 7); PHYT250, supplemented with 250 mg/head/d (n = 8); PHYT500, supplemented with 500 mg/head/d (n = 8). Supplements were a formulated blend of turmeric, capsicum, and black pepper extract in a fat carrier. Treatments were applied 30 days pre-calving up to 60 days post-calving.

Concentrations of IgG in milk (Figure 8) were unaffected by treatment × day interaction (*p* = 0.79), or main effect of treatment (linear: *p* = 0.24; quadratic: *p* = 0.29) but were greater at weaning compared to d 45 and d 90 (*p* < 0.01).

### 3.3. Cow and Calf Performance

**Figure 9:**
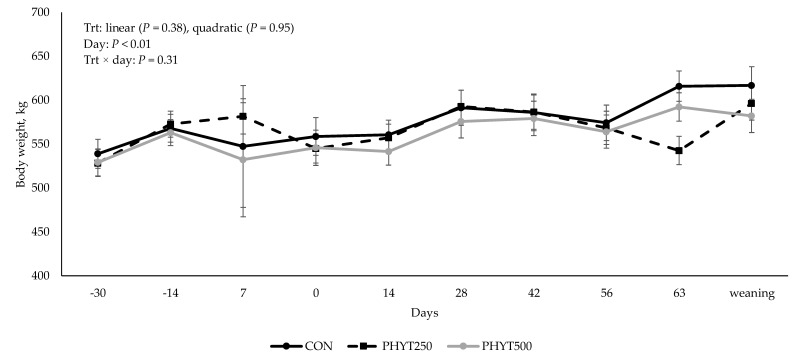
Effects of a botanical blend on cow body weight from 30 days pre-calving (d −30) up to weaning. Calving was considered d 0. Treatments were applied from 30 d pre-calving up to 60 d post-calving. CON, not supplemented (n = 7); PHYT250, supplemented with 250 mg/head/d (n = 8); PHYT500, supplemented with 500 mg/head/d (n = 8). Supplements were a formulated blend of turmeric, capsicum, and black pepper extract in a fat carrier.

**Figure 10:**
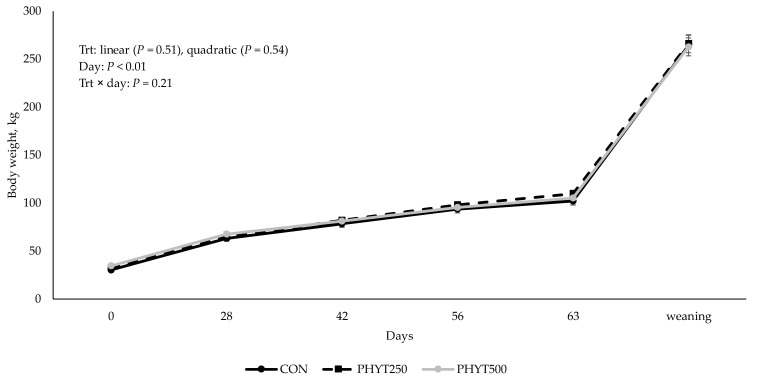
Effects of a botanical blend on calf body weight from birth (d 0) up to weaning. Treatments were applied to dams—cows received treatments from 30 d pre-calving up to 60 d post-calving. CON, not supplemented (n = 7); PHYT250, supplemented with 250 mg/head/d (n = 8); PHYT500, supplemented with 500 mg/head/d (n = 8). Supplements were a formulated blend of turmeric, capsicum, and black pepper extract in a fat carrier.

**Figure 11:**
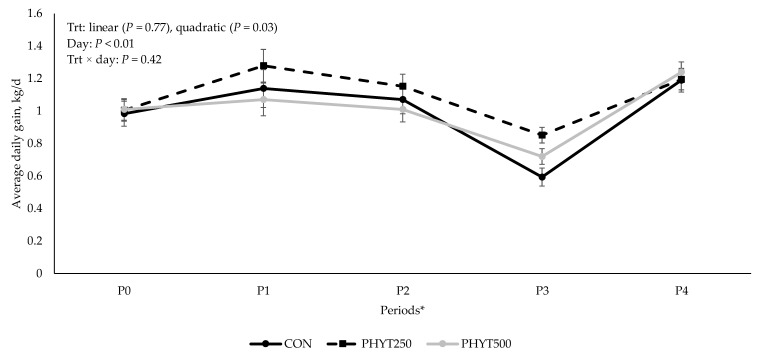
Effects of a botanical blend on calf average daily gain. * Period 0 (P0): birth to d 28. Period 1 (P1): d 28 to d 42. Period 2 (P2): d 42 to d 56. Period 3 (P3): d 56 to d 63. Period 4 (P4): d 63 to weaning. Treatments were applied to dams—cows received treatments from 30 d pre-calving up to 60 d post-calving. CON, not supplemented (n = 7); PHYT250, supplemented with 250 mg/head/d (n = 8); PHYT500, supplemented with 500 mg/head/d (n = 8). Supplements were a formulated blend of turmeric, capsicum, and black pepper extract in a fat carrier.

No treatment × day interaction (*p* = 0.31), nor linear (*p* ≥ 0.38) or quadratic (*p* ≥ 0.54) responses were observed for cow and calf body weight (Figure 9 and Figure 10). However, as expected, a significant effect of day (*p* < 0.01) was observed for both cow and calf body weight. A quadratic response was observed for calf ADG (*p* = 0.03; Figure 11), where CON (0.99 ± 0.03 kg/d) was less than PHYT250 (1.10 ± 0.03 kg/d), and intermediate values were observed for PHYT500 (1.01 ± 0.03 kg/d). Further, ADG was also affected by period, where the lowest ADG were observed for P3 (d 56 to d 63 of age) compared to all others.

## 4. Discussion

This research is the first to report the effects of a botanical supplement (blend of turmeric, capsicum, and pepper extracts) pre- and post-calving in beef cows on colostrum and milk quality, and yield, passive transfer of immunity, and cow–calf growth. Our findings reveal dose-dependent effects on several parameters, including colostrum composition, concentration of IgM in colostrum, concentrations of IgG and IgA in calf serum, and calf performance. These results indicate that PHYT may have the potential to enhance colostrum quality, improve calf immune responses, and increase ADG by influencing nutrient partitioning in the mammary gland and immunological and physiological processes in the offspring.

Colostrum consumption is essential for neonatal calves, providing the nutrients and antibodies required for survival and healthy development. Although colostrum and milk share some similarities, their distinct compositions reflect colostrum’s critical role in delivering passive immunity and promoting rapid neonatal growth [12]. The significance of colostrum cannot be overstated, given its critical role in supporting a calf’s immune status [13]. Colostrum production starts approximately 5 weeks pre-partum, with a peak of transfer of IgG1 from the maternal circulation to colostrum happening at 3 weeks prior to calving [14,15], which is the hallmark of colostrum formation. Therefore, in this study, we started applying our treatments between 4 and 5 weeks prior to calving (30 ± 6 days), to guarantee we would be within the critical timeline of colostrogenesis. Herein, colostrum production (d 0) was not affected by PHYT supplementation (average production of the left rear quarter = 403.69 mL); however, as discussed in detail in the following paragraphs, the nutrient composition and concentration of immunoglobulins were positively affected by PHYT. The colostrum production of the cows in this study was greater than reported by previous research [16,17]. Those authors reported an average colostrum production in the left rear quarter of 376.5 [16] and 293.95 [17]. However, both studies evaluated first-calf heifers, while in this study, we worked with second-calf cows, which may explain the greater values.

The observed trends in colostrum protein percentages suggest a link between PHYT supplementation and nitrogen metabolism. The quadratic tendency observed, with protein levels being increased in the PHYT500 group, may indicate that this level of supplementation stimulated protein synthesis during colostrogenesis, thus enhancing protein transfer to colostrum [18]. This suggests a dose-dependent response and highlights the need to further investigate the mechanisms by which PHYT influences protein synthesis or transfer to colostrum.

We observed a linear increase in fat content in both colostrum and milk for the PHYT-supplemented groups compared to the control. Fat concentration was significantly greater in colostrum for the supplemented groups, and tended to be greater in milk. Possibly, one of the oleoresins—or the combination of oleoresins—modified ruminal fermentation parameters, improving the production of acetate. Acetate is a key precursor for fatty acid synthesis in mammary glands, which may explain the dose-dependent effect of PHYT on colostrum and milk fat. One study reported increased milk fat in sows fed a proprietary phytogenic blend [19], while another observed decreased milk fat in sows fed oregano essential oils [20]. Research on the impact of botanical supplementation on fat content in milk and colostrum across species, especially in beef cows, is limited. Further investigation into the components and mechanisms of action of PHYT is necessary to clarify its role in enhancing milk fat content and its broader effects.

A possible explanation for the observed increase in calf serum IgA levels is turmeric’s ability to enhance IgA production. In a study, rats fed curcumin (a compound found in turmeric) had greater concentrations fecal IgA while on a high-fat diet, a condition that typically increases the risk of gut inflammation [21]. This suggests that turmeric may help mitigate inflammation and enhance mucosal immunity by increasing IgA levels. One proposed mechanism for this effect is turmeric’s interaction with the toll-like receptor 4 (TLR4) pathway, which plays a key role in the innate immune response to pathogens [22]. Turmeric is theorized to dimerize the TLR4 receptor, reducing its pro-inflammatory signaling, lowering cytokine production, and ultimately decreasing inflammation [22]. Additionally, in a heat stress study, curcumin supplementation in sheep was shown to increase serum IgA and IgM [23]. Although the available sources do not directly examine the effects of turmeric supplementation on calf health, the evidence from related studies provides a potential explanation for the observed results. Further, turmeric’s ability to modulate immune responses and reduce inflammation through mechanisms such as TLR4 pathway interaction suggests that it could play a role in enhancing immune function in cattle.

A tendency was observed for colostrum IgA concentrations to decrease as the level of turmeric supplementation increased, while, as previously mentioned, serum IgA concentrations tended to increase. This contrasting pattern suggests a potential shift in IgA allocation between serum and colostrum as a result of supplementation. These findings underscore the need for further research to better understand the mechanisms driving these effects and to clarify the implications of turmeric supplementation on immune function and colostrum composition in cow–calf production systems. The observed tendency for elevated IgG levels in calf serum when cows were fed the botanical supplement can be attributed to several potential mechanisms. As highlighted before, curcumin increased fecal IgA levels in rats under inflammatory conditions, suggesting that curcumin may broadly enhance Ig production, including IgG [21]. Additionally, Lee et al. (2011) observed that a blend of capsicum and turmeric oleoresins stimulated immune cell activity in chickens, including T cells, indicating enhanced adaptive immunity, which is critical to produce IgG antibodies [24]. T cells, specifically helper T cells, are crucial for activating B cells, which produce antibodies, by providing signals through direct interaction and cytokines [25]. While these studies focused on other species, the immune-stimulating properties of these compounds are likely to extend to IgG production in cattle.

A significant quadratic effect was observed for calf ADG, with the PHYT250 group showing the greatest ADG, the CON group the lowest, and PHYT500 displaying intermediate values. While body weights for cows and calves were unaffected, the enhanced ADG in the PHYT250 group suggests potential benefits at this dosage. It is worth mentioning that all calves (both male and female) were managed under the same conditions; thus, we assume that any performance differences observed reflect treatments applied to the dams. The ADG reported herein for the PHY250 calves (1.10 ± 0.03 kg/d) was greater than the average 0.95 to 1.04 kg/d reported for nursing beef calves [26]. As the price paid to producers depends on the weight of the calves at weaning, it is of paramount importance to identify managerial strategies that can improve average daily gain. Previous research on botanical feed additives in feedlot cattle showed mixed outcomes. Brand et al. [27] reported improved ADG but reduced dressing percentage with a botanical blend, while Rivaroli et al. [28] observed no effects on ADG or final BW in bulls supplemented with essential oils. Piran Filho et al. [4] observed increased carcass weights and a trend for greater BW with a proprietary botanical supplement. Conversely, Maia Ribeiro et al. [29] noted no changes in ADG, feed efficiency, or health outcomes in steers fed a proprietary blend of capsicum, clove, and garlic essential oils. Despite these findings, the effects of botanical additives in gestating/lactating beef cows and their offspring remain unexplored. Variability among supplements because of unique formulations further complicates comparisons, highlighting the need for more targeted research in this area.

## 5. Conclusions

In summary, supplementation with a botanical blend of turmeric, capsicum, and pepper extracts linearly increased fat content in colostrum and IgA concentrations in calf serum. Further, PHYT supplementation pre- and post-calving positively affected calf ADG. These findings suggest that both doses tested in this study (250 mg/d and 500 mg/d of PHYT) can efficiently enhance nutrient supply and immune support for neonatal beef calves. However, the optimal dose should be determined based on the specific goals and economic considerations of each operation. Further research is warranted to fully elucidate the biological significance and economic benefits of botanical additives in beef production, as they may represent a valuable tool for improving herd health and sustainably.

## 6. Patents

Provisional patent for this work has been filed in the United States.

## Figures and Tables

**Table 1:** Chemical composition and proportion of experimental diets.

	Experimental Diets	
Item ^1^	Pre-Calving	Post-Calving
Ingredients, % DM		
Corn silage	71	70
Grass hay	22	-
Dried distiller grains plus solubles	7	-
Alfalfa hay	-	30
Chemical composition, g/kg DM		
Organic matter	943.4	939.7
Crude protein	90.9	110.1
Ether extract	23.9	19.9
Neutral detergent fiber	449.9	417.6
Non-fiber carbohydrates	378.8	392.0

^1^ All values on dry matter basis. Diets were formulated according to NASEM (2016) requirements for gestating and lactating beef cows. Cows were individually fed commercial mineral and vitamin supplement formulated to supply and/or exceed NASEM (2016) requirements.

**Table 2:** Effects of supplementing a botanical blend in beef cows on colostrum and transition milk composition and volume on the first four days post-calving.

Item	Treatment ^1^	Days				TRT Average	SEM ^2^	*p*-Value			
		0	1	2	3			Linear	Quadratic	Day	TRT × Day
Fat, %	CON	3.81	3.10	3.46	2.77	3.29 ^x^					
	PHYT250	5.14	3.92	4.40	3.44	4.23 ^y^	0.77	0.05	0.19	0.50	0.26
	PHYT500	4.32	3.71	2.86	5.8	4.17 ^y^					
	day average	4.42	3.58	3.58	4.00						
Protein, %	CON	14.65	6.97	5.03	4.56	7.80					
	PHYT250	13.77	6.19	4.94	4.40	7.33	0.59	0.36	0.09	<0.01	0.16
	PHYT500	15.7	7.23	5.42	4.64	8.24					
	day average	14.71 ^a^	6.79 ^b^	5.13 ^c^	4.53 ^d^						
Other solids, %	CON	4.13	4.7	4.91	5.07	4.71					
	PHYT250	4.28	4.35	4.68	5.09	4.60	0.14	0.16	0.89	<0.01	0.28
	PHYT500	4.25	4.4	4.67	4.77	4.52					
	day average	4.22 ^d^	4.48 ^c^	4.76 ^b^	4.98 ^a^						
Lactose, %	CON	2.88	3.67	3.96	4.14	3.66					
	PHYT250	2.98	3.34	3.75	4.19	3.57	0.15	0.21	0.96	<0.01	0.45
	PHYT500	3.01	3.36	3.69	3.87	3.48					
	day average	2.96 ^d^	3.45 ^c^	3.79 ^b^	4.07 ^a^						
Colostrum, mL	CON	358.57	343.57	384.29	355.00	360.36					
(left rear quarter)	PHYT250	457.50	200.00	272.50	351.25	320.31	60.93	0.48	0.33	0.25	0.42
	PHYT500	395.00	385.00	460.00	443.75	420.94					
	day average	403.69	309.52	372.26	383.33						

^1^ Treatments: CON, not supplemented; PHYT250, supplemented with 250 mg/head/d; PHYT500, supplemented with 500 mg/head/d. Supplements were a formulated blend of turmeric, capsicum, and black pepper extract in a fat carrier. ^2^ Average SEM for the treatment × day interaction (for all days CON, n = 7; PHYT250, n = 8; PHYT500, n = 8). ^a–d^ Means within row with different superscripts differ significantly (*p* ≤ 0.05). ^x,y^ Means within column with different superscripts differ significantly (*p* ≤ 0.05)

**Table 3:** Effects of supplementing a botanical blend pre- and post-calving to beef cows on milk composition and volume at 45 and 90 d post-calving and at weaning.

Component	Treatment ^1^	Days			TRT Average	SEM ^2^	*p*-Value			
		45	90	Weaning			Linear	Quadratic	Day	TRT × Day
Fat, %	CON	4.07	3.66	3.79	3.84					
	PHYT250	3.95	3.87	4.33	4.05	1.04	0.07	0.56	0.85	0.83
	PHYT500	4.68	4.82	4.64	4.71					
	day average	4.23	4.12	4.26						
Protein, %	CON	3.19	3.18	3.70	3.36					
	PHYT250	3.26	3.13	3.97	3.45	0.18	0.95	0.29	<0.01	0.14
	PHYT500	3.27	3.13	3.69	3.36					
	day average	3.24 ^b^	3.15 ^c^	3.79 ^a^						
MUN mg/100 g	CON	16.67	15.97	18.11	16.92					
	PHYT250	15.25	16.61	16.83	16.23	1.16	0.06	0.73	0.08	0.23
	PHYT500	12.75	16.70	15.53	14.99					
	day average	14.89	16.43	16.82						
Other solids, %	CON	5.67	5.60	4.55	5.27					
	PHYT250	5.65	5.61	4.44	5.23	0.27	0.55	0.97	<0.01	0.30
	PHYT500	5.63	5.26	4.66	5.18					
	day average	5.65 ^a^	5.49 ^b^	4.55 ^c^						
Lactose, %	CON	4.76	4.71	3.59	4.35					
	PHYT250	4.74	4.71	3.48	4.31	0.28	0.67	0.96	<0.01	0.36
	PHYT500	4.73	4.42	3.71	4.28					
	day average	4.74 ^a^	4.61 ^b^	3.59 ^c^						
Volume, mL	CON	5925.00	4501.67	3491.67	4639.44					
	PHYT250	6072.50	6321.25	4456.25	5616.67	604.06	0.12	0.28	<0.01	0.50
	PHYT500	6345.00	5922.00	4426.25	5564.58					
	day average	6114.17 ^a^	5581.81 ^a^	4124.72 ^b^						

^1^ Treatment: CON, not supplemented; PHYT250, supplemented with 250 mg/head/d; PHYT500, supplemented with 500 mg/head/d. Supplements were a formulated blend of turmeric, capsicum, and black pepper extract in a fat carrier. ^2^ Average SEM for the treatment × day interaction (for all days CON, n = 7; PHYT250, n = 8; PHYT500, n = 8). ^a–c^ Means within row with different superscripts differ significantly (*p* ≤ 0.05).

## Data Availability

The data presented in this study are available in the main article.

## Abbreviations

The following abbreviations are used in this manuscript:

ADG	Average daily gain
BW	Body weight
DDGS	Dried distiller grains with solubles
DM	Dry matter
Ig	Immunoglobulin
IgA	Immunoglobulin A
IgG	Immunoglobulin G
IgM	Immunoglobulin M
MUN	Milk urea nitrogen
RID	Radial immunodiffusion
TLR4	Toll-like receptor 4
TMR	Total mixed ration
